# Prognostic impact of a new score using neutrophil-to-lymphocyte ratios in the serum and malignant pleural effusion in lung cancer patients

**DOI:** 10.1186/s12885-017-3550-8

**Published:** 2017-08-22

**Authors:** Yong Seok Lee, Hae-Seong Nam, Jun Hyeok Lim, Jung Soo Kim, Yeonsook Moon, Jae Hwa Cho, Jeong-Seon Ryu, Seung Min Kwak, Hong Lyeol Lee

**Affiliations:** 1 Department of Obstetrics and Gynecology, The Catholic University of Korea, Seoul, 06591 South Korea; 2 Division of Pulmonology, Department of Internal Medicine, Inha University Hospital, Inha University School of Medicine, 27, Inhang-ro, Jung-gu, Incheon, 22332 South Korea; 3 Department of Laboratory Medicine, Inha University Hospital, Inha University School of Medicine, Incheon, 22332 South Korea

**Keywords:** Lung cancer, Malignant pleural effusion, Neutrophil-to-lymphocyte ratio, Prognostic factor, Serum

## Abstract

**Backgrounds:**

Various studies have reported that the neutrophil-to-lymphocyte ratio in the serum (sNLR) may serve as a cost-effective and useful prognostic factor in patients with various cancer types. However, no study has reported the prognostic impact of the NLR in malignant pleural effusion (MPE). To address this gap, we investigated the clinical impact of NLR as a prognostic factor in MPE (mNLR) and a new scoring system that use NLRs in the serum and MPE (smNLR score) in lung cancer patients.

**Methods:**

We retrospectively reviewed all of the patients who were diagnosed with lung cancer and who presented with pleural effusion. To maintain the quality of the study, only patients with malignant cells in the pleural fluid or tissue were included. The patients were classified into three smNLR score groups, and clinical variables were investigated for their correlation with survival.

**Results:**

In all, 158 patients were classified into three smNLR score groups as follows: 84 (53.2%) had a score of 0, 58 (36.7%) had a score of 1, and 16 (10.1%) had a score of 2. In a univariate analysis, high sNLR, mNLR, and increments of the smNLR score were associated with shorter overall survival (*p* < 0.001, *p* = 0.004, and *p* < 0.001, respectively); moreover, age, Eastern Cooperative Oncology Group performance status (ECOG PS), histology, M stage, hemoglobin level, albumin level, and calcium level were significant prognostic factors. A multivariable analysis confirmed that ECOG PS (*p* < 0.001), histology (*p* = 0.001), and smNLR score (*p* < 0.012) were independent predictors of overall survival.

**Conclusions:**

The new smNLR score is a useful and cost-effective prognostic factor in lung cancer patients with MPE. Although further studies are required to generalize our results, this information will benefit clinicians and patients in determining the most appropriate therapy for patients with MPE.

**Electronic supplementary material:**

The online version of this article (doi:10.1186/s12885-017-3550-8) contains supplementary material, which is available to authorized users.

## Background

Malignant pleural effusions (MPEs), which are diagnosed based on the identification of malignant cells in the pleural fluid or on pleural biopsy, represent an advanced malignant disease that is associated with high morbidity and mortality; these characteristics preclude the possibility of a curative treatment approach. Despite major advances in cancer treatment over the past two decades, the median survival time (MST) following a diagnosis of MPE depends on the origin of the primary tumor as well as its histological type and stage, and usually ranges from 4 to 12 months. Lung cancer patients with MPE have the shortest survival times. For this reason, the revised staging system for lung cancer upstaged the presence of MPE from T4 to M1a [1–5].

These patients with advanced lung cancer experienced an improvement in their quality of life and received less aggressive care at the end of their lives with early palliative care than with the current standard of care [6]. In addition, palliative care is favorable in terms of medical cost savings. These results suggest an urgent need to develop more useful and cost-effective clinical prognostic factors that may help to select the most appropriate care and to minimize inconvenience for the remainder of the patients’ lives. However, many studies have reported various molecular biomarkers that may predict the prognoses of cancer patients, but technical factors and excessive costs still preclude their clinical use [7].

Recently, a meta-analysis comprising 100 studies reported that an elevated neutrophil-to-lymphocyte ratio in the serum (sNLR), which is one of several systemic inflammatory markers, is associated with an adverse overall survival (OS) many types of solid tumors, and thus the sNLR may serve as a useful and cost-effective prognostic factor [8]. However, no study on the NLR of MPE (mNLR) has been reported thus far. Only one study showed that high neutrophil levels in MPE were significantly associated with adverse OS of patients with MPE [9]. These data suggest that the mNLR, like the sNLR, may serve as a new prognostic factor in patients with MPE.

Accordingly, we questioned whether the mNLR has a prognostic impact in patients with MPE. To address this question, we reviewed different cell counts of MPE in lung cancer patients who presented with pleural effusion and investigated the prognostic impact of the mNLR. Furthermore, we investigated the clinical impact of a new scoring system that incorporates the NLRs in the serum and MPE (smNLR score).

## Methods

### Study population

We retrospectively reviewed all patients diagnosed with lung cancer who presented with pleural effusion between 2002 and 2010 at Inha University Hospital. To maintain the quality of the study, only patients with malignant cells confirmed in the pleural fluid or on pleural biopsy were included in the study. To identify malignant cells in effusion fluid and/or pleural biopsy tissue, a conventional cytology examination and/or histological analyses were performed independently. With respect to the conventional cytologic examination, 5 ~ 10 ml of effusion fluid obtained by diagnostic thoracentesis was centrifuged at 2500 rpm for 10 min, and a minimum of two thin smears were prepared from the sediment. One smear was air-dried and stained with Leishman-Giemsa stain, and the other smear was immediately fixed in 95% alcohol and stained with Papanicolaou stain according to the hospital pathology laboratory’s standard protocol. With respect to the histological analyses, tissue specimens obtained during pleural biopsy were processed after formalin fixation; sections were then stained with hematoxylin-eosin dye**.** The stages of all patients were defined according to the seventh edition of the TNM classification system [4]. The study protocol was approved by the Institutional Review Board of Inha University Hospital. Informed consent was waived because of the retrospective nature of the study.

### Data collection

Baseline prognostic clinical and laboratory variables were collected retrospectively from the electronic medical record system. Patient-related variables included age, gender, smoking status, Eastern Cooperative Oncology Group performance status (ECOG PS), and the serum levels of hemoglobin, albumin, lactate dehydrogenase (LDH), and calcium at diagnosis. The tumor-related variables consisted of histology and stage. Finally, the treatment variables were classified into two subgroups, as follows: active treatments, including systemic chemotherapy and/or radiation therapy to the lung, and supportive treatments, including supportive care, refusal of treatment, and radiation therapy to metastatic sites for symptomatic palliation.

The NLRs were obtained by dividing the absolute number of neutrophils by the number of lymphocytes in the complete blood count of the serum at diagnosis and in the total cell count of MPE obtained during diagnostic thoracentesis.

### The new score using NLRs of the serum and MPE (smNLR score)

The optimal cutoff values for the sNLR and the mNLR were determined using maximally selected rank statistics [10, 11]. Maximally selected rank statistics were calculated using R software, version 3.03 (The R Foundation for Statistical Computing, Vienna, Austria; http://www.r-project.org) and the ‘maxstat’ package. According to the cutoff values for the sNLR and the mNLR, we defined the smNLR score as follows: patients in whom both the sNLR (≥3.85) and the mNLR (≥1.36) were elevated were assigned a score of 2. Patients in whom only one of the two NLR values was elevated were assigned a score of 1. Patients in whom neither the sNLR nor mNLR valuess was elevated were assigned a score of 0.

### Statistical analysis

OS was measured as an outcome and was estimated from the time of diagnosis until death as a result any cause. Only two patients died of causes other than lung cancer. The distribution of variables according to the smNLR score was assessed by χ^2^ tests. Survival analyses were performed using the Kaplan-Meier method and log-rank test. Potential predictors of survival were entered into univariate Kaplan-Meier models and compared using the log-rank test. Factors with a prognostic association in the univariate analysis were entered into a multivariate Cox regression model (forward sequential method) to determine their independent effects. The results of the Cox regression modeling are presented as hazard ratios and associated 95% confidence intervals. Variables with *p*-values less than 0.05 were considered statistically significant. All analyses were performed using the IBM SPSS statistical software package version 19.0 (SPSS, Chicago, IL, USA).

## Results

### Patient characteristics

In all, 158 patients underwent diagnostic thoracentesis. Eighty-one of these patients also underwent parietal pleural biopsy. A diagnosis of MPE was confirmed by both cytology and biopsy in 62 patients, by cytology alone in 84 patients, and by biopsy alone in 12 patients. No causes of infection, such as bacteria, tuberculosis, or viruses, were identified in the blood, sputum, or MPE of any of the cases. The baseline characteristics of the study population are summarized in Table 1. The median age of the patients was 68 years (range: 32–89), and 81 patients were male (51.3%). The majority of patients were former or current smokers (53.8%), had an ECOG PS of 0–1 (59.5%) and exhibited an adenocarcinoma histology (85.4%). At the time of diagnosis, 51.9% of patients had distant metastases in other organs outside the lung (M1b). The percentages of patients who received supportive and active treatments were 46.8 and 53.2%, respectively. Seventy-six patients in the active treatment group received chemotherapy; out of these, 70 patients received platinum-based doublet chemotherapy and 6 patients received gemcitabine monotherapy; the other 8 patients received radiation therapy to the lung. All of the patients died. Survival data was collected from the electronic medical record system and the Korean Ministry of Security and Public Administration.

**Table 1:** Baseline characteristics according to the new score, which uses the neutrophil-to-lymphocyte ratios in serum and malignant pleural effusion (smNLR score) in lung cancer patients

Variable	Total	smNLR score			*P* value
	*n* = 158	0 (*n* = 84)	1 (*n* = 58)	2 (*n* = 16)	
Age, years					0.246
< 65 ≥ 65	60 (38.0) 98 (62.0)	37 (44.0) 47 (56.0)	18 (31.0) 40 (69.0)	5 (31.3) 11 (68.7)	
Sex					0.167
Male Female	81 (51.3) 77 (48.7)	40 (47.6) 44 (52.4)	35 (60.3) 23 (39.7)	6 (37.5) 10 (62.5)	
Smoking habit					0.391
Current + Former Never	85 (53.8) 73 (46.2)	43 (51.2) 41 (48.8)	35 (60.3) 23 (39.7)	7 (43.8) 9 (56.2)	
ECOG PS					0.001
0–1 2–4	94 (59.5) 64 (40.5)	61 (72.6) 23 (27.4)	28 (48.3) 30 (51.7)	5 (31.3) 11 (68.7)	
Histology					0.023
ADC SQC Others SCC	135 (85.4) 9 (5.7) 5 (3.2) 9 (5.7)	77 (91.6) 4 (4.8) 0 (0.0) 3 (3.6)	48 (82.8)) 2 (3.4) 4 (6.9) 4 (6.9)	10 (62.5) 3 (18.8) 1 (6.2) 2 (12.5)	
M Stage					0.002
M1a M1b	76 (48.1) 82 (51.9)	51 (60.7) 33 (39.3)	21 (36.2) 37 (63.8)	4 (25.0) 12 (75.0)	
Treatment					0.056
Supportive Active	74 (46.8) 84 (53.2)	32 (38.1) 52 (61.9)	32 (55.2) 26 (44.8)	10 (62.5) 6 (37.5)	
Hemoglobin, g/dL^a^					0.170
< 12 ≥ 12	59 (37.3) 99 (62.7)	27 (32.1) 57 (67.9)	23 (39.7) 35 (60.3)	9 (56.2) 7 (43.8)	
Albumin, g/dL^a^					<0.001
< 3.1 ≥ 3.1	30 (19.0) 128 (81.0)	7 (8.3) 77 (91.7)	15 (25.9) 43 (74.1)	8 (50.0) 8 (50.0)	
LDH, IU/L^a^					0.261
≤ 211 > 211	76 (48.1) 82 (51.9)	45 (53.6) 39 (46.4)	23 (39.7) 35 (60.3)	8 (50.0) 8 (50.0)	
Calcium, mg/dL^a^					0.140
≤ 10.8 > 10.8	156 (98.7) 2 (1.3)	83 (98.8) 1 (1.2)	58 (100) 0 (0.0)	15 (93.7) 1 (6.3)	
sNLR					<0.001
< 3.85 ≥ 3.85	87 (55.1) 71 (44.9)	84 (100) 0 (0.0)	3 (5.2) 55 (94.8)	0 (0.0) 16 (100)	
mNLR					<0.001
< 1.36 ≥ 1.36	139 (88.0) 19 (12.0)	84 (100) 0 (0.0)	55 (94.8) 3 (5.2)	0 (0.0) 16 (100)	

Data in parentheses are percentages

*Abbreviations*: *ECOG PS* Eastern Cooperative Oncology Group performance status, *ADC* adenocarcinoma, *SQC* squamous cell carcinoma, *SCC* small cell carcinoma, *LDH* lactate dehydrogenase, *sNLR* neutrophil-to-lymphocyte ratio of serum, *mNLR* neutrophil-to-lymphocyte ratio of malignant pleural effusion

^a^Dichotomized by cutoff of normal value

### Clinical factors associated with the new smNLR score

All patients were classified into one of three smNLR score groups as follows: 84 (53.2%) had a score of 0, 58 (36.7%) had a score of 1, and 16 (10.1%) had a score of 2. The clinical and laboratory factors associated with the three smNLR score groups are shown in Table 1. The mean ± standard deviation of the sNLR and the mNLR were 4.91 ± 3.99 (range: 1.21–24.43) and 0.64 ± 1.74 (range: 0.00–17.20), respectively. Age, gender, smoking status, treatment, hemoglobin, LDH, and calcium were not significantly different among the three groups. However, besides the sNLR (*p* < 0.001) and the mNLR (*p* < 0.001), ECOG PS (*p* = 0.001), histologic type (*p* = 0.023), M stage (*p* = 0.002), and the level of albumin (*p* < 0.001) exhibited significant differences among the three groups.

### Types of NLRs and overall survival

**Fig. 1:**
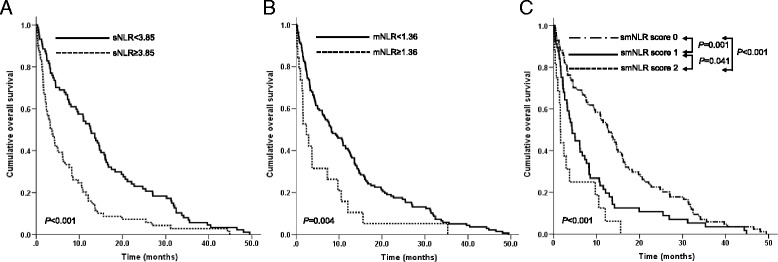
Overall survival of the total study population according to (**a**) the neutrophil-to-lymphocyte ratio in serum (sNLR), (**b**) the neutrophil-to-lymphocyte ratio in malignant pleural effusion (mNLR), and (**c**) the new score that encompasses the neutrophil-to-lymphocyte ratios in the serum and malignant pleural effusion (smNLR score)

The MST of all patients was 7.7 months (95% confidence interval: 5.3 ~ 10.1). The results of the univariate analyses of individual baseline variables are listed in Table 2. The following variables were associated with shorter OS: age ≥ 65 (*p* < 0.001), ECOG PS 2–4 (*p* < 0.001), non-adenocarcinoma histologic type (*p* < 0.001), M1b stage (*p* = 0.002), palliative treatment (*p* = 0.007), anemia (*p* = 0.004), hypoalbuminemia (*p* < 0.001), and hypercalcemia (*p* < 0.001). All types of NLRs (sNLR, mNLR and smNLR score) were also significant prognostic factors in the univariate analysis, as follows: high sNLR and mNLR were associated with a shorter OS (sNLR <3.85 vs ≥3.85, MST 12.6 vs 3.6 months, respectively, *p* < 0.001, Fig. 1a; and mNLR <1.36 vs ≥1.36, MST 8.3 vs 2.4 months, respectively, *p* = 0.004, Fig. 1b). An increment in the smNLR score was also associated with a shorter OS (smNLR score 0 vs 1 vs 2, MST 12.6 vs 4.4 vs 1.6 months, respectively, *p* < 0.001, Fig. 1c).

**Table 2:** Univariate and multivariate analyses of the factors that are predictive of overall survival in all patients (*n* = 158)

Variable	Univariate analysis			Multivariate analysis		
	MST, mo	95% CI	*P* value	HR	95% CI	*P* value
Age, years			<0.001			
< 65 ≥ 65	13.6 4.3	11.1–16.2 2.7–5.9				
Sex			0.103			
Male Female	5.0 9.5	2.8–7.3 6.5–12.5				
Smoking habit			0.052			
Current + Former Never	4.5 9.7	2.1–6.9 6.8–12.6				
ECOG PS			<0.001			<0.001
0–1 2–4	13.6 2.4	11.8–15.4 1.5–3.3		reference 3.88	2.66–5.64	
Histology			<0.001			0.001
ADC SQC Others SCC	8.5 3.7 1.5 3.1	5.7–11.3 0.0–11.0 0.0–4.2 0.8–5.4		reference 2.07 4.79 2.25	1.01–4.26 1.86–12.36 1.09–4.65	0.048 0.001 0.029
M Stage			0.002			
M1a M1b	10.9 4.3	6.9–14.9 2.6–6.0				
Treatment			0.007			
Supportive Active	3.2 10.9	1.9–4.5 8.1–13.7				
Hemoglobin, g/dL^a^			0.004			
< 12 ≥ 12	3.3 9.2	0.7–5.9 6.5–12.0				
Albumin, g/dL^a^			<0.001			
< 3.1 ≥ 3.1	2.6 9.5	1.1–4.0 6.7–12.3				
LDH, IU/L^a^			0.119			
≤ 211 > 211	10.6 4.5	8.1–13.1 3.1–6.0				
Calcium, mg/dL^a^			<0.001			
≤ 10.8 > 10.8	7.7 1.0	5.1–10.2				
sNLR			<0.001			
< 3.85 ≥ 3.85	12.6 3.6	9.7–15.6 2.2–5.0				
mNLR			0.004			
< 1.36 ≥ 1.36	8.3 2.4	5.1–11.4 0.5–4.3				
smNLR score			<0.001			0.012
0 1 2	12.6 4.4 1.6	9.7–15.6 2.7–6.0 1.4–1.8		reference 1.37 2.36	0.95–1.98 1.30–4.28	0.090 0.005

*Abbreviations*: *MST* median survival time, *mo* month, *CI* confidence interval, *HR* hazard ratio, *ECOG PS* Eastern Cooperative Oncology Group performance status, *ADC* adenocarcinoma, *SQC* squamous cell carcinoma, *SCC* small cell carcinoma, *sNLR* neutrophil-to-lymphocyte ratio of serum, *mNLR* neutrophil-to-lymphocyte ratio of malignant pleural effusion, *smNLR score* score using neutrophil-to-lymphocyte ratios of serum and malignant pleural effusion

^a^Dichotomized by cutoff of normal value

Individual variables that were analyzed in the univariate analyses were entered into the multivariate Cox model, irrespective of their significance. A multivariate analysis revealed the following prognostic variables to be independent predictors of shorter OS (Table 2): ECOG PS 2–4 (*p* < 0.001), non-adenocarcinoma histologic type (*p* = 0.001), and increments in the smNLR score (*p* < 0.012). On the contrary, a multivariate analysis apart from the smNLR score showed that age > 65 (*p* = 0.046), ECOG PS 2–4 (*p* < 0.001), non-adenocarcinoma histologic type (*p* = 0.02), and an sNLR ≥3.85 (*p* = 0.007) were independent predictors of shorter OS (Additional file 1: Table S1).

## Discussion

In this study, we found that the sNLR, mNLR and smNLR scores are significant prognostic factors for adverse OS in lung cancer patients with MPE. Furthermore, the new smNLR score was found to be a more significant independent prognostic factor than the sNLR alone, which has been reported as a prognostic factor in patients with various types of cancer [8, 12–19]. The smNLR score is readily calculated, inexpensive, and universally available in clinical settings from the different cell counts in the serum and MPE. Therefore, the smNLR score could potentially be an attractive and ideal prognostic factor that predicts the survival of patients with MPE, which may provide valuable additional prognostic information for doctors and patients. To the best of our knowledge, the present study is the first report on the prognostic impact of the smNLR score.

Inflammation has been reported to play an important role in different stages of tumorigenesis and is now considered a hallmark of cancer [20, 21]. In recent years, many studies have investigated the most commonly used measures of the systemic inflammatory response, such as C-reactive protein, the Glasgow Prognostic Score, cytokines, and leucocytes, and their potential prognostic impact in cancer patients [22–26]. In addition, some systematic reviews have shown that the numbers of leucocyte subtypes, specifically the neutrophil and lymphocyte counts, are objective parameters with the ability to express the severity of the systemic inflammatory response in patients with cancer [8, 12, 14]. These studies have reported that an elevated sNLR has a consistent effect on adverse OS among patients with various solid tumors and the tumor stages. Recently, a study on the prediction of survival in patients with MPE showed that the sNLR is a significant prognostic factor in a multivariable analysis and that the LENT prognostic score (pleural fluid LDH, ECOG PS, the sNLR, and tumor type) has significantly higher accuracy than ECOG PS alone [5]. Therefore, the sNLR has enormous potential as a readily available and inexpensive biomarker. As shown by the present multivariate analysis, with the exception of the new smNLR score (Additional file 1: Table S1), the prognostic impact of the sNLR in lung cancer patients with MPE was consistent with the results of previous studies.

The mechanisms that are associated with high sNLR and poor outcome in cancer patients remain unclear. One potential mechanism that has been proposed involves the interactions between the tumor and host cells such as leucocytes due to an inflammatory response. As an inflammatory response, neutrophilia inhibits the immune system via the suppression of the cytolytic activity of immune cells such as lymphocytes and activated T cells. In addition, tumor-infiltrating lymphocytes have been reported to exert a positive effect on the survival of cancer patients. Taken together, the prognostic impact of the sNLR may be due to the association of a high sNLR with inflammation [8, 14, 15, 27]. These potential mechanisms suggest that the mNLR could also have a potential prognostic impact in patients with MPE, but since the cause of MPE is still unclear, its impact may be ascribed to the hematogenous direct spread of tumor cells to the pleura [2, 3]. In our study, we found that a higher mNLR was adversely associated with survival in lung cancer patients with MPE. In addition, the smNLR score was a more significant independent prognostic factor compared with the sNLR or mNLR alone. Another merit of the smNLR score is that it may be calculated based on tests that are routinely performed for patients with MPE in a variety of clinical settings.

One study reported that, compared with patients who received standard care, early palliative care of patients with advanced lung cancer was associated with improvement in the quality of life, less aggressive end-of-life care, and longer survival [6]. Therefore, in patients with advanced cancer, simpler, more useful, and more cost-effective clinical prognostic factors at presentation may help to not only individualize treatment strategies but also to minimize inconvenient and unnecessary aggressive treatment. Although further studies are required to apply our results to a clinical setting, the smNLR score may provide better information to clinicians and patients, which might enable them to select the most appropriate therapy for patients with MPE.

This study has several limitations and strengths. First, this study is a single-center study with a relatively small sample size. The findings were also not validated in an independent series of patients, which limits our ability to generalize our findings. However, approximately 15% of lung cancer patients have been reported to present with pleural effusion; moreover, the diagnostic sensitivity of pleural fluid cytological and/or closed pleural biopsy analysis usually ranges from 40 to 87%. Generally, the more invasive approach of thoracoscopy might be indicated when pleural cytology and/or biopsy are negative and MPE is still suspected [2, 3]. All patients in our study were confirmed to have malignant cells in the pleural fluid or on pleural biopsy, and not all of our cases required the more invasive procedure. We believe that this inclusion criterion is causative of the size of the sample and the improvement in study quality. The present results using the mNLR may provide new implications for prognostic factors in patients with MPE, but validation is needed in another independent group to generalize our results. Second, the retrospective nature of this study is associated with limitations that pertain to selection, exclusion, and recall bias. However, although all items related to pleural fluid of all included patients who were diagnosed MPE were not available, the mNLR was available in all patients. Furthermore, general prognostic factors related to advanced lung cancer were considered in detail in this study [28]. Recently, molecular biomarkers, which were not considered in our study, have been demonstrated to be important prognostic factors in lung cancer patients. The test for epidermal growth factor receptor (EGFR) mutations has significant prognostic relevance in lung cancer patients, particularly in those with advanced adenocarcinoma [29, 30]. Unfortunately, the EGFR mutation test for MPE was not available at our institution during the time the present study was conducted. Finally, the NLR is a nonspecific variablethat may be influenced by concurrent conditions such as infections, inflammation, and medications. This limitation has been observed in most studies of the sNLR [8]. However, to maintain the quality of the present study, we included only patients in whom malignant cells were identified in the pleural fluid or on pleural biopsy, and none of the included cases had identifiable causes of infection, such as bacteria, tuberculosis, or viruses, in the blood, sputum, or MPE. We also determined the optimal cutoff values for the sNLR and the mNLR using maximally selected rank statistics [10, 11] to maintain the objectivity of our study.

## Conclusions

In summary, high sNLR and mNLR values are associated with adverse OS, and the smNLR score is a more significant independent prognostic factor than the sNLR or the mNLR alone in lung cancer patients with MPE. To the best of our knowledge, this is the first study that has investigated the prognostic impact of the smNLR score in patients with MPE. This study suggests that the smNLR score may act as a simple, useful, and cost-effective prognostic factor in patients with MPE. Furthermore, these results may serve as the cornerstone of further research into the mNLR in the future. This information will benefit clinicians and patients in determining the most appropriate therapy for patients with MPE.

## Additional file

Additional file 1: Table S1. Multivariate analyses of the factors that are predictive of overall survival in all patients apart from the new score, which use the neutrophil-to-lymphocyte ratios in the serum and malignant pleural effusion. (DOCX 16.8 kb)

## Abbreviations

ADC: Aadenocarcinoma
CI: Cconfidence interval
CRP: C-reactive protein
ECOG PS: Eastern Cooperative Oncology Group performance status
EGFR: Epidermal growth factor receptor
HR: Hazard ratio
LDH: Lactate dehydrogenase
mNLR: Neutrophil-to-lymphocyte ratio of malignant pleural effusion
mo: Month
MPE: Malignant pleural effusion
MST: Median survival time
OS: Overall survival
SCC: Small cell carcinoma
smNLR score: Score using neutrophil-to-lymphocyte ratios of serum and malignant pleural effusion
sNLR: Neutrophil-to-lymphocyte ratio of the serum
SQC: Squamous cell carcinoma
