# Supplementary material for: Prognostic impact of a new score using neutrophil-to-lymphocyte ratios in the serum and malignant pleural effusion in lung cancer patients
Source: BMC Cancer. 2017 Aug 22;17:557. doi: 10.1186/s12885-017-3550-8 (PMC5567470; doi:10.1186/s12885-017-3550-8)
Supplement: Additional file 1: Table S1. — Multivariate analyses of the factors that are predictive of overall survival in all patients apart from the new score, which use the neutrophil-to-lymphocyte ratios in the serum and malignant pleural effusion. (DOCX 16.8 kb) [file 12885_2017_3550_MOESM1_ESM.docx]

**Supplementary Table 1.** Multivariate analyses of the factors that are predictive of overall survival in all patients apart from the new score, which use the neutrophil-to-lymphocyte ratios of serum and malignant pleural effusion

| Variable | Multivariate analysis | | |
| --- | --- | --- | --- |
|  | HR | 95% CI | *P* value |
| Age, years  < 65  ≥ 65 | reference  1.45 | 1.01-2.09 | 0.046 |
| ECOG PS  0-1  2-4 | reference  3.55 | 2.42-5.20 | <0.001 |
| Histology  ADC  SQC  Others  SCC | reference  2.25  3.67  2.09 | 1.12-4.50  1.42-9.51  1.01-4.34 | 0.003  (0.022)  (0.007)  (0.047) |
| sNLR  < 3.85  ≥ 3.85 | reference  1.62 | 1.14-2.30 | 0.007 |

*Abbreviations:* HR, hazard ratio; CI, confidence interval; ECOG PS, Eastern Cooperative Oncology Group performance status; ADC, adenocarcinoma; SQC, squamous cell carcinoma; SCC, small cell carcinoma; sNLR, neutrophil-to-lymphocyte ratio of serum
